# Bone conditioned media (BCM) improves osteoblast adhesion and differentiation on collagen barrier membranes

**DOI:** 10.1186/s12903-016-0230-z

**Published:** 2016-07-04

**Authors:** Masako Fujioka-Kobayashi, Jordi Caballé-Serrano, Dieter D. Bosshardt, Reinhard Gruber, Daniel Buser, Richard J. Miron

**Affiliations:** Department of Cranio and Maxillofacial Surgery, University of Bern, Bern, Switzerland; Department of Oral Surgery and Stomatology, Laboratory of Oral Cell Biology, University of Bern, Freiburgstrasse 7, Bern, 3010 Switzerland; Robert K. Schenk Laboratory of Oral Histology, University of Bern, Bern, Switzerland; Department of Oral Biology, University of Vienna, Vienna, Austria; Department of Oral Surgery and Stomatology, University of Bern, Bern, Switzerland; Department of Periodontology, College of Dental Medicine, Nova Southeastern University, Fort Lauderdale, Florida USA

**Keywords:** Bone conditioned media, Guided bone regeneration, Bone grafting, Barrier membranes, Growth factors

## Abstract

**Background:**

The use of autogenous bone chips during guided bone regeneration procedures has remained the gold standard for bone grafting due to its excellent combination of osteoconduction, osteoinduction and osteogenesis. Recent protocols established by our group have characterized specific growth factors and cytokines released from autogenous bone that have the potential to be harvested and isolated into bone conditioned media (BCM). Due to the advantageous osteo-promotive properties of BCM, the aims of the present study was to pre-coat collagen barrier membranes with BCM and investigate its effect on osteoblast adhesion, proliferation and differentiation for possible future clinical use.

**Methods:**

Scanning electron microscopy (SEM) was first used to qualitative assess BCM protein accumulation on the surface of collagen membranes. Thereafter, undifferentiated mouse ST2 stromal bone marrow cells were seeded onto BioGide porcine derived collagen barrier membranes (control) or barrier membranes pre-coated with BCM (test group). Control and BCM samples were compared for cell adhesion at 8 h, cell proliferation at 1, 3 and 5 days and real-time PCR at 5 days for osteoblast differentiation markers including Runx2, alkaline phosphatase (ALP), osteocalcin (OCN) and bone sialoprotein (BSP). Mineralization was further assessed with alizarin red staining at 14 days post seeding.

**Results:**

SEM images demonstrated evidence of accumulated proteins found on the surface of collagen membranes following coating with BCM. Analysis of total cell numbers revealed that the additional pre-coating with BCM markedly increased cell attachment over 4 fold when compared to cells seeded on barrier membranes alone. No significant difference could be observed for cell proliferation at all time points. BCM significantly increased mRNA levels of osteoblast differentiation markers including ALP, OCN and BSP at 5 days post seeding. Furthermore, barrier membranes pre-coated with BCM demonstrated a 5-fold increase in alizarin red staining at 14 days.

**Conclusion:**

The results from the present study suggest that the osteoconductive properties of porcine-derived barrier membranes could be further improved by BCM by significantly increasing cell attachment, differentiation and mineralization of osteoblasts in vitro. Future animal testing is required to fully characterize the additional benefits of BCM for guided bone regeneration.

## Background

The use of barrier membranes has commonly been utilized in guided tissue regeneration (GTR) and guided bone regeneration (GBR) procedures to selectively direct the growth and repopulation of bone and periodontal tissues from faster growing soft tissues [1, 2]. A variety of biomaterials have been used to achieve this role with much of their development occurring in the early 1980s [3, 4]. Such materials should ideally facilitate cell recruitment and attachment of both soft and hard tissue cells, increase cell proliferation, and induce differentiation towards their specific tissue types [5–8]. While GTR and GBR techniques were first introduced using bio-inert, non-resorbable expanded polytetrafluoroethylen membranes (ePTFE), more recently resorbable collagen membranes have been used due to the commonly reported problems of ePTFE including the requirement for a second surgery to remove the barrier, increased patient morbidity, additional surgical costs and increased possibility of bacterial colonization [9, 10]. The second generation of barrier membranes (resorbable membranes) are commonly fabricated from porcine collagen I and III and have demonstrated similar clinical results when compared to ePTFE without the requirement of a second surgery [11–15].

Third generation barrier membrane protocols now include their combination with a variety of growth factors including bone morphogenetic proteins (BMPs), platelet-derived growth factor (PDGF), enamel matrix derivative (EMD) or fibroblast growth factor-2 (FGF2) [16–20]. While additional growth factor utilization do incur further costs to the patient, numerous reports have demonstrated their effectiveness in select cases where limitations in defect anatomy could additionally necessitate the use of potent growth factor to facilitate wound healing and regeneration of tissues [16–20].

Recently our laboratory has been highly interested in discovering the key elements of autogenous bone grafts that make them so favorable for bone augmentation procedures [21]. Initial experiments revealed that harvesting with a variety of different surgical techniques could significantly alter the survival of bone cells as well as their release of growth factors [22, 23]. These preliminary studies revealed a tight regulation over the control of factors being released to the surrounding media as well as their potential to either induce or resorb surrounding bone.

The second series of experiments focused mainly on harvesting these factors in something now termed ‘bone conditioned medium’ (BCM) and discovering if these factors alone could influence cell behaviour [22, 24–28]. Here, the primary focus was to test the efficacy of BCM on a variety of cell types demonstrating that the BCM medium serves as a strong inducer of the TGF-beta cell activity responsible for the differentiation of various cell types. Simply stated, fresh bone chips contain viable cells, mainly osteocytes capable of controlling bone remodeling through a variety of growth factor proteins that induce gene expression in mesenchymal progenitor cells [24–26, 28]. Therefore, the possibility exists that collection released growth factors from autogenous bone (bone-conditioned media) over a period of time followed by re-delivery to subsequent cells/tissues may potentially be a source of autogenous growth factor delivery specific for ideal bone regeneration.

To date, no study has yet to investigate the influence of BCM in combination with collagen barrier membranes on osteoblast behaviour. As common growth factor delivery vehicles include collagen barrier membranes due to their advantageous adsorption of growth factors, the aim of the present study was to investigate the combination of barrier membranes pre-coated with BCM as a possible growth factor source derived from autogenous source. The effect of BCM in combination with barrier membranes was tested on osteoblast adhesion, proliferation and differentiation in vitro.

## Methods

### BCM and barrier membranes

Bone was obtained from adult pigs (Metzgerei Balsiger, Wattenwil, Switzerland) and harvested from the buccal-sided mandibular cortical bone with a “bone scraper” (Hu-Friedy, Rotterdam, Netherlands) and placed into sterile plastic dishes as previously described [26]. An in vivo ethical approval was not necessary as the animals were euthanized at the local butcher for non-scientific reasons and thereafter the bone samples were immediately collected and transported for use in this study. BCM was harvested by placing 10 mL of culture media with 10 g of autogenous bone chips. After 24 h of incubation at 37 °C in a humidified atmosphere and 5 % carbon dioxide, BCM was filtered sterile and kept frozen at −80 °C and thawed during experimental cell seeding as previously described [26]. For all in vitro cell experiments, membranes were loaded with 20 % BCM at time point 0 for 5 min. Membranes were incubated for 5 min with BCM based on our group’s previous investigation that found that an adsorption plateau of growth factors occurred roughly around 5 min [29]. Afterwards, routine cell culture media was utilized. A porcine derived collagen barrier membrane (BioGide, Geistlich, Switzerland) was utilized as the material of choice for the present study due to our laboratories previously handling with its use [30]. For in vitro experiments, barrier membranes were cut and placed in the bottom of 24 well dishes and thereafter coated with BCM for 5 min prior to cell seeding.

### Cell culture system

Undifferentiated mouse cell-line ST2 stromal bone marrow cells were used for this study.

Cells were detached from the tissue culture plastic using trypsin solution. During cell seeding for differentiation experiments, α-MEM medium (Invitrogen, Basel, Switzerland) was supplemented with 50 μg/ml ascorbic acid (Invitrogen) and 2 mM β-glycerophosphate (Invitrogen) to promote osteoblast differentiation as previously described [23]. Osteoblasts were seeded at a density of 10,000 cells in 24 well culture plates for cell adhesion and proliferation experiments and 50,000 cells per well in 24 well dishes for real-time PCR and alizarin red experiments. For experiments lasting longer than 5 days, medium was replaced twice weekly.

### Adhesion and proliferation assay

Osteoblasts were seeded in 24-well plates at a density of 10,000 cells per well either control barrier membranes or barrier membranes + BCM. Cells were quantified using fluorescent MTS assay (Invitrogen) at 8 h for cell adhesion and 1, 3 and 5 days for cell proliferation as previously described [30]. At desired time points, cells were washed with phosphate buffered solution (PBS) and quantified using a fluorescence plate reader (Infinite 200, Tecan Group Ltd. Männedorf, Switzerland). Experiments were performed in triplicate with three independent experiments for each condition. Means and standard deviations (SE) were calculated, and the statistical significance of differences was examined by student *t*-test between both groups for cell adhesion (*, *p* values < 0.05 was considered significant). Data for cell proliferation were analyzed for statistical significance using two-way analysis of variance with Bonferroni test (*, *p* values < 0.05 was considered significant).

### Real-time PCR for differentiation markers

Real-time RT-PCR was used to investigate the expression of genes encoding osteoblast differentiation markers. Total RNA was isolated using High Pure RNA Isolation Kit (Roche, Switzerland) at 5 days for osteoblast differentiation markers. Primer and probe sequences for genes encoding alkaline phosphatase (ALP), runt-related transcription factor 2 (Runx2), osteocalcin (OCN), bone sialoprotein (BSP) and glyceraldehyde 3-phosphate dehydrogenase (GAPDH) were fabricated with Primer sequences according to Table 1. Real-time RT-PCR was performed using Roche Master mix and quantified on an Applied Biosystems 7500 Real-Time PCR Machine. All samples were assayed in duplicate with 3 independent experiments were performed. The ∆∆Ct method was used to calculate gene expression levels normalized to GAPDH values and calibrated to control samples. Means and standard deviations (SE) were calculated, and the statistical significance of differences was examined by student *t*-test between both groups (*, *p* values < 0.05 was considered significant).

**Table 1:** PCR primers for genes encoding Runx2, ALP, BSP, OCN and GAPDH

Gene	Primer sequence
mRUNX2 F	agggactatggcgtcaaaca
mRUNX2 R	ggctcacgtcgctcatctt
mBSP F	gcatcgaagagtcaaaatag
mBSP R	ttcttctccattgtcttctc
mALP F	ggacaggacacacacacaca
mALP R	caaacaggagagccacttca
mOCN F	cagacaccatgaggaccatc
mOCN R	ggactgaggctctgtgaggt
mGAPDH F	aggtcggtgtgaacggatttg
mGAPDH R	tgtagaccatgtagttgaggtca

### Alizarin red staining

Alizarin red staining was performed to determine the presence of extracellular matrix mineralization. After 14 days, cells were fixed in 96 % ethanol for 15 min and stained with 0.2 % alizarin red solution (Sigma Aldrich) in water (pH 6.4) at room temperature for 1 h as previously described [23]. Alizarin red staining was performed using images captured on a Nikon D610 camera with a Heerbrugg M400 ZOOM microscope (WILD HEERBRUGG, Switzerland). Image J software was used to quantify data using set parameters for colour intensity staining of red using color threshold including parameters for hue, saturation and brightness. The same threshold values were used for all analyzed. Means and standard deviations (SE) were calculated, and the statistical significance of differences was examined by one-way analysis of variance with Bonferroni test (*, *p* values < 0.05 was considered significant).

## Results

### Influence of BCM on surface coating, osteoblast adhesion and proliferation

**Fig. 1:**
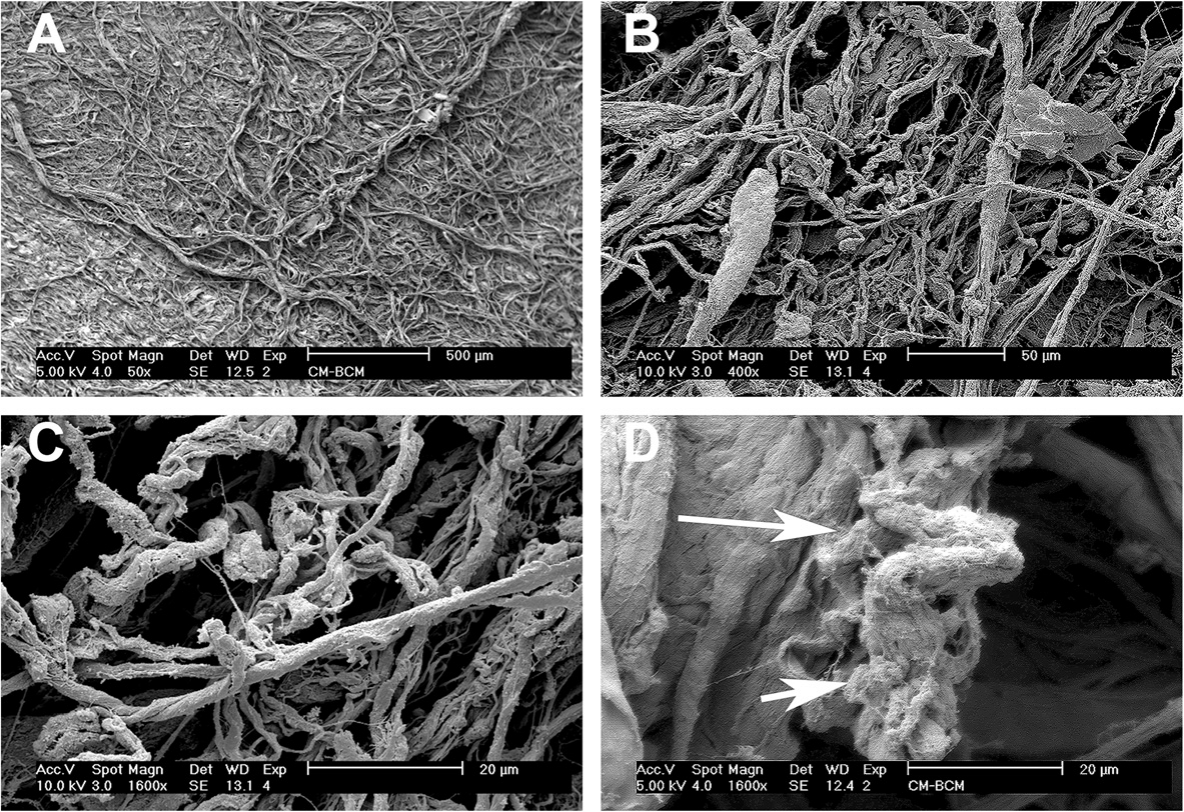
SEM images of control collagen barrier membranes at (**a**) 50X and (**b**) 400 X magnification. The additional coating of BCM onto collagen membranes increased surface protein accumulation (**d**, arrows 1600X magnification) when compared to control samples (**c**, 1600X magnification)

**Fig. 2:**
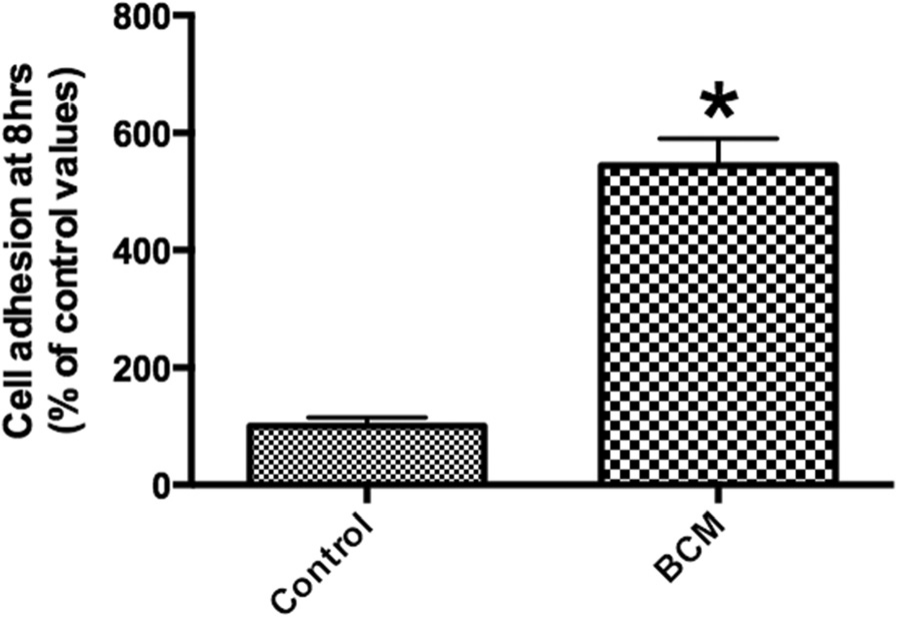
Attachment assay of ST2 osteoblasts seeded on control barrier membranes in comparison to membranes + BCM. The combination of barrier membranes with BCM significantly upregulated osteoblast attachment onto barrier membranes at 8 h post seeding (* denotes significant difference, *p* < 0.05)

**Fig. 3:**
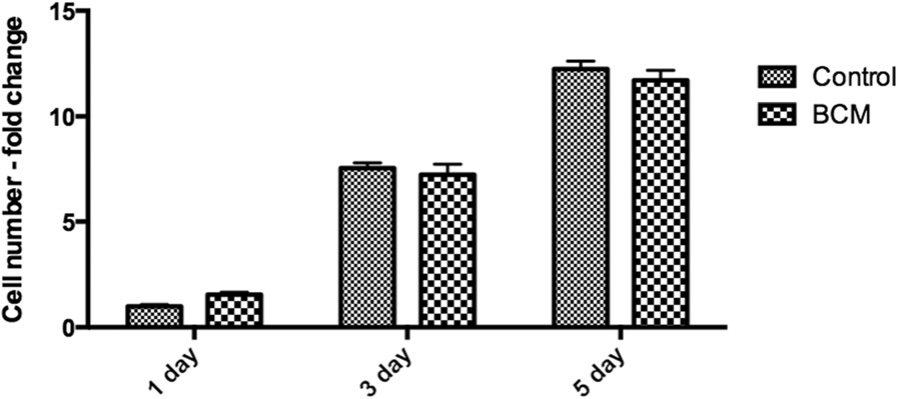
Proliferation assay of ST2 osteoblasts seeded on control barrier membranes and barrier membranes with BCM at 1, 3 and 5 days post seeding. No significant difference could be observed between treatment samples

First, SEM was utilized to investigate the influence of pre-coating BCM onto collagen membranes for variations in surface topography/protein coating (Fig. 1). While control barrier membranes demonstrated many collagen fibrils found on their surface (Fig. 1a, b), the additional coating with BCM revealed the presence of accumulated proteins found on the surface of the barrier membrane (Fig. 1d, arrows), when compared to control samples at high magnification (Fig. 1c). Thereafter, the effects of BCM were investigated on cell adhesion of osteoblasts to barrier membranes. It was found that coating BCM onto barrier membranes significantly enhanced over 5 fold the number of attached osteoblasts 8 h post seeding when compared to uncoated control barrier membranes (Fig. 2). The effects of BCM however demonstrated no effect on osteoblast proliferation as quantified by an MTS assay at 1, 3 and 5 days post seeding (Fig. 3).

### Influence of BCM on osteoblast gene expression and mineralization

**Fig. 4:**
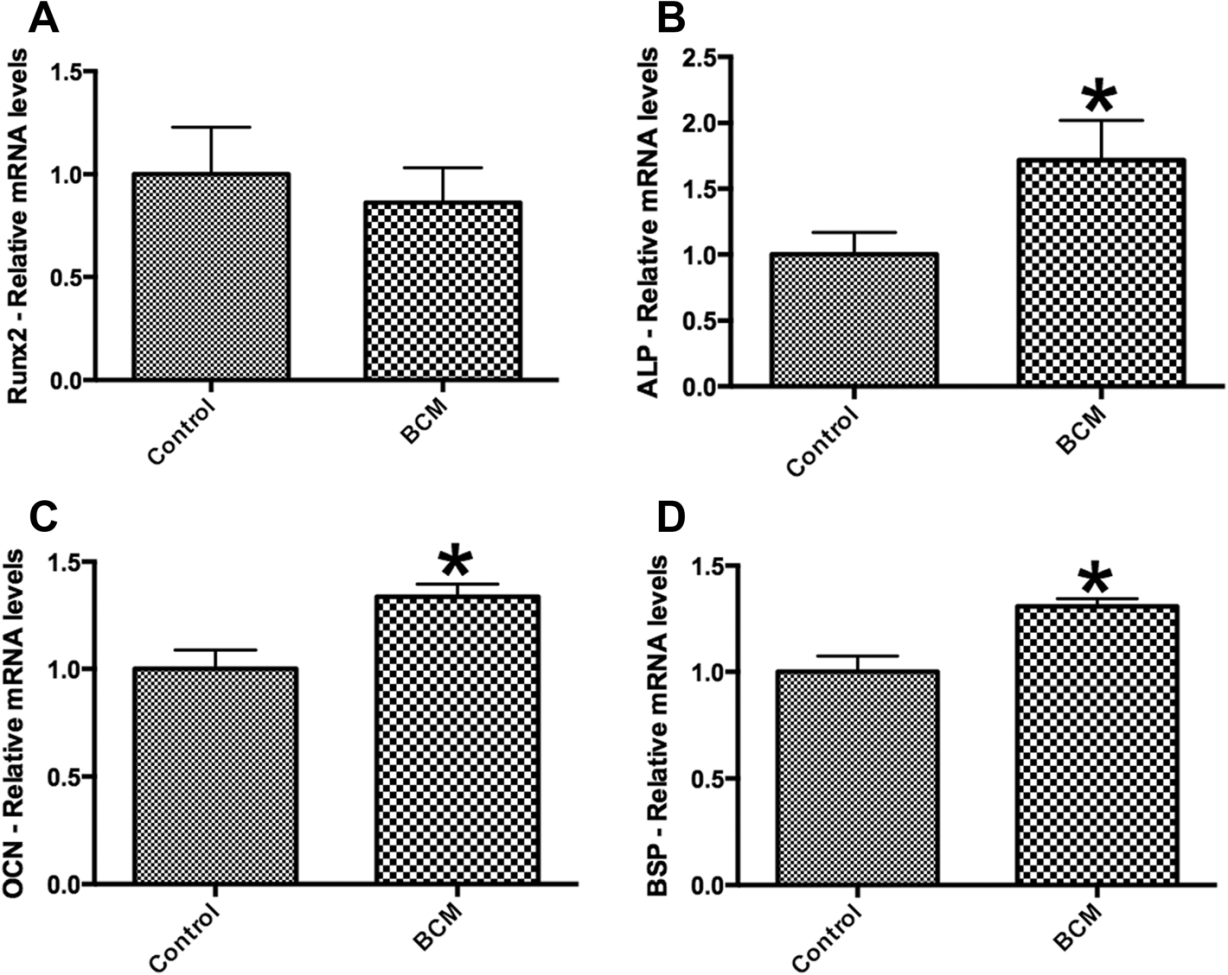
Real-time PCR of osteoblasts seeded on control barrier membranes and barrier membranes pre-coated with BCM for genes encoding (**a**) Runx2, (**b**) alkaline phosphatase (ALP), (**c**) osteocalcin (OCN) and (**d**) bone sialoprotein (BSP) at 5 days post seeding (* denotes significant difference, *p* < 0.05)

**Fig. 5:**
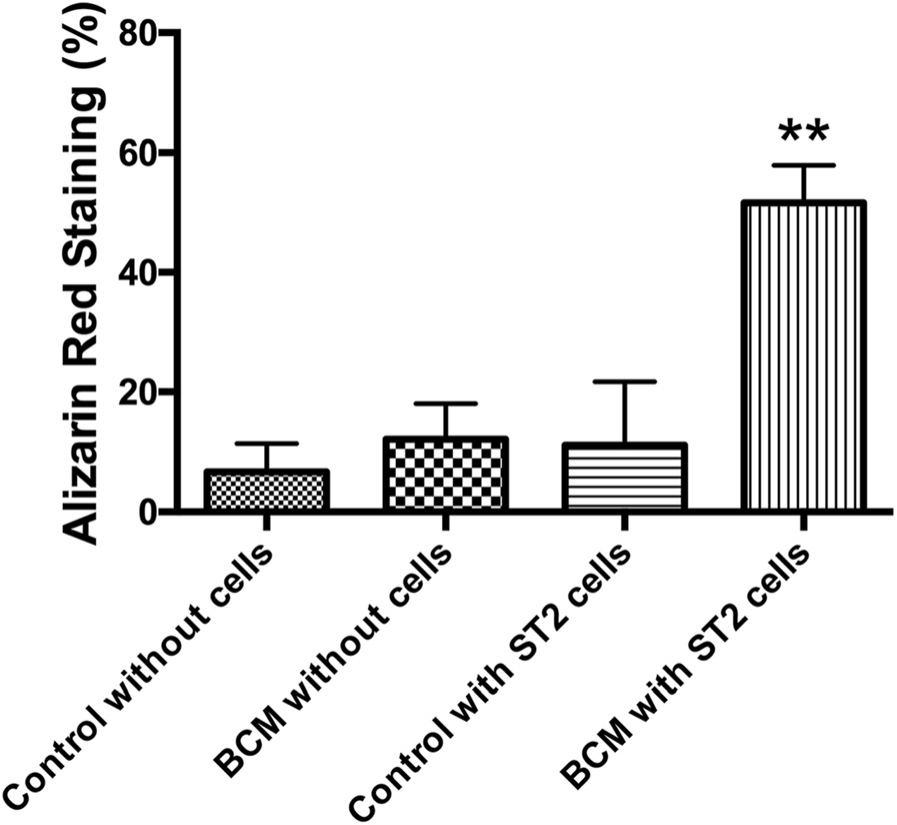
Alizarin red staining at 14 days on (1) control barrier membranes without ST2 cells, (2) barrier membranes pre-coated with BCM without ST2 cells, (3) ST2 cells on control barrier membranes and (4) ST2 cells on barrier membranes pre-coated with BCM. Semi-quantified evaluation of alizarin red staining. Pre-coating collagen membranes with BCM significantly increased alizarin red staining as assessed by colour thresholding software (* denotes significantly higher compared with the other modalities, *p* < 0.05)

The addition of BCM to collagen barrier membranes was then investigated on osteoblast differentiation. Real-time PCR for genes encoding Runx2 demonstrated no influence of BCM when combined with a barrier membrane (Fig. 4a). In contrast, pre-coating BCM onto barrier membranes significantly increased mRNA levels of ALP (Fig. 4b), OCN (Fig. 4c) and BSP (Fig. 4d) at 5 days post seeding (*p* < 0.05). Furthermore, alizarin red staining was used to quantify in vitro mineralization (Fig. 5). It was found that barrier membranes pre-coated with BCM revealed a 5-fold increase in alizarin red staining when compared to control barrier membranes as well as barrier membranes pre-coated with BCM without cells (negative control) (Fig. 5).

## Discussion

The aim of the present study was to investigate the potential benefits of pre-coating BCM onto barrier membranes on the in vitro cell behaviour of bone-forming osteoblasts. To date, no study has investigated the potential of BCM on osteoblast differentiation when combined with a biomaterial. Therefore, in light of these recent findings by our group demonstrating the effects of BCM, it was hypothesized that BCM could be used as a potential autogenous growth factor source derived specifically from bone tissues for bone regeneration.

Several studies by our group have extensively investigated in vitro cell behavior in response to BCM. In these studies, cortical bone chips obtained from porcine mandibles were collected using a bone scraper and incubated in cell culture medium for 24 h to allow growth factor accumulation [31]. Initial experiments were designed to investigate contained proteins within BCM via proteomics analysis. It was found that more than 40 different growth factors and extracellular matrix molecules were found released from bone chips into BCM; known factors capable of inducing osteoblast and osteoclast activity [32, 33]. Analysis of the entire genome for cells cultured with BCM indicated that BCM serves as a strong inducer of TGF-beta dependent pathways by significantly altering the expression of IL11, IL33, ADM, NOX4, PRG4, and PTX3 [34]. Based on these novel findings demonstrating the effects of BCM on TGF-beta dependent pathways in gingival fibroblasts [34], it was hypothesized that BCM could potentially induce the activity of osteoblast progenitor cells towards mineral-producing mature osteoblasts, specifically when coated in combination with a collagen barrier membrane.

It was found that BCM significantly upregulated the adhesion of osteoblasts to barrier membranes over 5 fold when compared to non-coated membranes (Fig. 2). As it is known that cells need to bind to biomaterials through extra-cellular matrix proteins, the present study hypothesized that certain proteins found in BCM contain cell adhesion domains able to enhance cell attachment of mesenchymal cells. Future investigation regarding cell-protein binding sites in response to BCM remains to be characterized. Furthermore, analysis of various integrin-binding domains could prove strategic to further characterize the responsible proteins found in BCM capable of facilitating cell attachment to biomaterials that are pre-coated with growth factors from an autogenous bone source.

Results from the present study also confirm that BCM is able to significantly increase gene expression of BSP and OCN, known markers of osteoblast differentiation. While the significant increase was observed at 1.5 times control values, alizarin red staining revealed a 5-fold enhancement in staining intensity when collagen membranes were pre-coated with BCM. Thus, it may be hypothesized that the accumulated proteins contained within BCM may possess the potential to speed in vivo bone formation due to its influence on cell behavior in vitro. Future investigation regarding the potential use of BCM for a clinical setting remains to be investigated.

Two key questions remain unanswered from the present study. First, the stock BCM utilized in the present study was collected from cell culture media with bone chips over a period of 24 h. The potential use of BCM for clinical application would require a markedly shorter time period for growth factor accumulation. An average implant procedure last roughly 30 to 60 min [35] and would ideally be the maximum time required to accumulate enough growth factors for possible use. Thus it remains to be investigated if the concentration obtained from this short period would be enough to maintain its influence on differentiation towards bone-forming osteoblasts. Furthermore, at presence it is unknown what effect the concentration of BCM might have on hard tissue formation in vivo. For instance, it is well known that recombinant proteins necessitate very higher concentrations in vivo when compared to in vitro studies [30]. It remains to be investigated what the ideal concentration for in vivo use and potentially additional methods to further concentrate BCM for further clinical utilization as an autogenous growth factor source specifically derived from bony tissues.

## Conclusion

The results from the present study demonstrate that pre-coating collagen barrier membranes with BCM significantly increased osteoblast attachment when compared to barrier membranes alone. Furthermore, pre-coating collagen membranes with BCM enhanced osteoblast differentiation and mineralization as assessed by real-time PCR and alizarin red staining. The combination of these findings suggests that BCM may be a valuable method to collect bone-derived growth factors from autogenous source for future biomaterial coating. Future experiments to determine the optimal culturing period, optimal carrier system as well as assessment of new bone formation in an animal model are now necessary to further investigate the clinical potential of BCM.

## Abbreviations

ADM, Adrenomedullin; ALP, alkaline phosphatase; BCM, bone conditioned media; BMP, bone morphogenetic protein; BSP, bone sialoprotein; EMD, enamel matrix derivative; ePTFE, expanded polytetrafluoroethylen membranes; FGF2, fibroblast growth factor 2; GAPDH, glyceraldehyde 3-phosphate dehydrogenase; GBR, guided bone regeneration; GTR, guided tissue regeneration; IL11, interleukin 11; IL33, interleukin 33; NOX4, NADPH oxidase 4; OCN, osteocalcin; PBS, phosphate buffered solution; PDGF, platelet derived growth factor; PRG4, Proteoglycan 4; PTX3, Pentraxin 3; Runx2, runt-related transcription factor 2; SEM, scanning electron microscopy; TGFB, transforming growth factor beta
